# Influence of freeze-dried acid whey addition on biogenic amines formation in a beef and deer dry fermented sausages without added nitrite

**DOI:** 10.5713/ajas.19.0011

**Published:** 2019-05-28

**Authors:** Anna D. Kononiuk, Małgorzata Karwowska

**Affiliations:** 1 Department of Meat Technology and Food Quality, University of Life Sciences in Lublin, ul. Skromna 8, 20-704 Lublin, Poland

**Keywords:** Biogenic Amines, Dry Cured Sausages, Fallow Deer, Beef, Acid Whey

## Abstract

**Objective:**

Aim of this study was to evaluate the influence of freeze-dried acid whey addition and the use a game meat (fallow deer) on a microbial content and the biogenic amines formation in dry fermented sausages.

**Methods:**

The experiment involved dry fermented sausages made in two variants from beef and from fallow deer. Each variant was divided into five groups: control (with a curing mixture), reference (with a sea salt), sample with a liquid acid whey and two samples with the addition of reconstituted freeze-dried acid whey in different concentrations. Changes in lactic acid bacteria (LAB), *Enterobacteriaceae* content and biogenic amines content were determined.

**Results:**

The microbial content changes suggest that addition of acid whey slightly affected LAB content in comparison with the control and reference sample, but the addition of freeze-dried acid whey resulted in a reduction of *Enterobacteriaceae* content in the sausages from fallow deer or a similar level in the beef sausages compared with the control and reference sample. Both changes in LAB and *Enterobacteriaceae* content were more evident in case of sausages made from fallow deer. Addition of acid whey (liquid and a higher amount of freeze-dried) and use of fallow deer meat to produce the sausages resulted in a significant reduction of total biogenic amines content.

**Conclusion:**

The addition of acid whey (liquid and higher amount of freeze-dried) resulted in a significant reduction of total biogenic amines content in dry fermented sausages made from fallow deer meat.

## INTRODUCTION

Biogenic amines (BA) are nitrogen compounds which are formed from amino acids through biochemical reactions catalysed by amino acid decarboxylases. They may be of endogenous origin (naturally occurring in animal cells) or exogenous enzymes produced by microorganisms [1]. The BA play important role in humans. They take part in regulation of a body temperature and a blood pressure, also are essential for maintaining the activities of metabolic, immunological, nervous and vascular systems. However, when large amounts of BA are digested or an organism is not able to catabolise unwanted BA, they can exert a toxic effect [1–3]. Moreover, some amines such as putrescine or cadaverine can also react with a nitrite and form potentially carcinogenic N-nitroso compounds [4]. BA occur in variety of foods, such as a fish, meat, fruits and vegetables. Food which has microbiological spoilage or has been prepared by a fermentative process is especially characterized by high levels of BA [5–7]. One of the important sources of dietary BA are fermented and dry-cured meat products. According to European Food Safety Agency (EFSA) Scientific Opinion the most frequently occurring BA in fermented and cured meat is a tyramine [8]. Other numerous occurring BA in meat products are cadaverine, putrescine and histamine [5,9].

Intensity of BA formation depends mainly on microbial growth, acidification and proteolysis. The formation of BA is the consequence of decarboxylation of amino acids, which mainly depends on activity of microbial decarboxylation enzymes. Substrates for this reaction are amino acids so degradation of protein into peptides and amino acids causes an increase in the content of precursors for BA. Finally, conditions during processing as a pH value, water activity or temperature can favour bacterial growth and microbial decarboxylase synthesis and activity [4,10]. Bover-Cid et al [11] in their paper investigated bacteria isolated from a fermented pork sausage for amino acid-decarboxylase activity. Their results suggested that the BA formation and decarboxylases activity are not associated to bacteria species but to the specific strains of bacteria. However, they suggested that mainly lactic acid bacteria (LAB) are associated with tyrosine formation, and to a lesser extent they also can contribute to the production of phenyletylamine, tryptamine, putrescine and cadaverine [11]. Moreover, the LAB are commonly occur and are used to prepare fermented foods [12].

The BA content in meat products may vary depending on factors during processing including type of meat and curing using nitrate or nitrite [13]. The use of ingredients to replace added nitrites is related to the need to assess their impact on the content of BA. The addition of acid whey allows the production of meat products without nitrite which have a similar or better physico-chemical and sensory properties than the conventional products with a curing salt [14,15]. The unique properties of acid whey in meat processing are mainly determined by the presence of lactic acid and whey microorganisms. Acid whey obtained from a traditional cottage cheese production is the source of large number of bacteria, which represent many species and strains. It is dominated LAB included to *Lactobacillus* strains [16]. Acid whey is very perishable and contains large amounts of water, which limits its use as an additive to sausage production. For this reason, an attempt was made to preserve and concentrate the acid whey obtained from the traditional cottage cheese production. Freeze-drying was chosen because of difficulties in drying acid whey with other methods due to its high lactic acid content [17]. Research by Papavasiliou et al [18] indicates that acid whey has cryoprotective properties. Thus, freeze-drying of acid whey should produce a liophylisate powder containing naturally occurring microorganisms which is protected against spoilage and can be reconstituted to the desired concentration [18].

However, the study [14,15] did not address an important safety-related aspect, formation of BA. Literature contains a little information about formation of BA in meat products from wild animals.

In this context, the aim of this study was to compare BA content between two types of sausages (with commonly used beef and the lesser used fallow deer). Additives were chosen to determine the influence of using acid whey as an alternative for nitrite, also the freeze-dried acid whey was used to study the effect of increased concentration of acid whey on BA formation.

## MATERIALS AND METHODS

### Fermented sausage production

The raw materials including beef meat, fallow deer meat and a beef tallow came from a breeding farm. The farm produce was certified as organic by a Polish certifying body according to the article 29(1) of Council Regulation (EC) No. 834/2007 on organic production and labeling of organic products. Additional ingredients used to produce sausages in this study included sea salt (CurodiMare, Apulia, Italy) and acid whey. According to the certificate, the sea salt used in the experiment contained no sodium nitrate and nitrate. Acid whey was obtained from the traditional cottage cheese production (Organic Farm Robert Janowski, Ludwinów, Poland) and contained 93.23%±0.22% of water. Acid whey was used in liquid form and lyophilized in laboratory freeze drier (Labconco Free-Zone, Kansas City, MO, USA). Table 1 presents characteristics of acid whey in liquid and freeze-drying form.

Dry cured sausages were manufactured in two variants from different types of the meat (fallow deer and beef). In both cases beef tallow was used as the fat raw material. The raw materials were chopping and ground through a plate with a mesh diameter 8 mm in a universal machine (MESKO-AGD, Skarżysko-Kamienna, Poland). After that five batches with different additives were prepared from beef and another five from fallow deer meat (the amount of additives is shown in Table 2): C, (a control sample) with the curing mixture (99.5% sea salt, 0.5% sodium nitrite); S, (a reference sample) with the sea salt; AW, with the liquid acid whey; LAW, with the reconstituted, freeze-dried acid whey; 2LAW, with the reconstituted double portion of freeze-dried acid whey. Every time the acid whey was reconstituted from the lyophylizate using saline solution. The meat, fat (in a ratio of 80:20 [w/w]) and the appropriate additives were mixed and stuffed into fibrous casings (95 mm). The sausages were put into a fermentation chamber and kept at 16°C until the weight loss was about 30%. The stuffing and sausages after ripening were used to analyze changes in LAB and *Enterobacteriaceae* content and BA content.

### Content of *Enterobacteriaceae* and lactic acid bacteria

Samples for microbiological analyses were taken after grinding and mixing the entire sample. All samples were taken in triplicate and carried out at Agrolab Polska Sp. z o. o. (Dęblin, Poland). The measurements were performed in accordance with ISO/IEC Standard 17025:2005 [19]. The results were calculated as a difference between content of bacteria at the beginning and end of process and were expressed as colony forming unit per 1 gram of product (cfu/g).

### Content of biogenic amines

The BA extraction process was carried out by homogenizing 10 g of each sausage sample with 100 mL of 10% trichloroacetic acid in a homogenizer (15,000 rpm, 1 min, IKA T25D, Staufen, Germany). The homogenate was extracted for 1 hour in fridge. Next the samples were centrifuged (5,000 rpm, 20 min, 4°C, MPW 350R, Warszawa, Poland). The supernatants were filtered through a Whatman filter No. 1, passed back through a 0.22 μm nylon filter (Alfachem, Lublin, Poland) and were stored at 4°C until analysis.

The analysis of BA was performed using an AAA 500 amino acid analyzer (Ingos, Prague, Czech Republic), equipped with an Ostion LG AAA8 ion-exchange column (3.6×100 μm, 8 μm). Separation was by a stepwise gradient elution using Na^+^/K^+^ citric buffers. Colorimetric detection was at 570 nm, after post-column derivatization of ninhydrine. Content of the BA (histamine, tyramine, putrescine, cadaverine, spermidine, agmatine, spermine) was determined with a reference to the amine standards, which were supplied by Ingos, Czech Republic. The BA concentrations were reported as mg/g of product.

### Data analysis

The experiment was carried out on two samples from each of the sausages in three replications. The results were expressed as mean±standard deviation. The Dell Statistica v.13 (data analysis software system) was used for the statistical treatment of the data. The main effects and interactions between means were calculated using the two-way analysis of variance method. The significant differences between subgroups have been specified based on post hoc Tukey’s procedure. Pearson’s correlation coefficient was calculated to measure the strength and direction of the relationship between studied variables. All statistical results were considered significant at p<0.05.

## RESULTS AND DISCUSSION

### Microbiological characteristic of sausages

The changes in LAB and *Enterobacteriaceae* content between the beginning and the end of processing of dry cured sausages are presented in Table 3. According to data presented in the Table 4, type of meat, additives used and the interaction between them had significant effect on microbial changes in dry fermented sausages (p<0.01, except that the effect of additives on changes in LAB content had a significance level of p<0.05). During the processing the content of LAB increased in all samples. Nevertheless, in the samples made from fallow deer the increase was clearly higher. In the sausages made from beef, changes in the LAB content are highly correlated with the subsequent applied additives (correlation coefficient = 0.87). Addition of acid whey (especially addition of double portion of acid whey) caused a significantly higher increase of LAB (p<0.05). In case of the sausages made from fallow deer that significant dependence was not observed (p>0.05). Only in the sample with the double portion of acid whey was the increase of LAB content on a similar level as in the control and reference samples. Such results could mean that addition of the higher amount of acid whey inserts more of the desired bacteria into the sausage stuffing. This is in accordance with results obtained by other authors, who observed a higher amount of LAB in samples with acid whey addition [20,21]. During the processing *Enterobacteriaceae* content decreased in the all samples. The largest decreases were observed in the curing sausage (C) from the beef meat and in the fallow deer sausages with the different addition of acid whey (AW, LAW, 2LAW). Only the *Enterobacteriaceae* content changes in the fallow deer sausages were indeed correlated with the subsequent additives (correlation coefficient = 0.64). However, changes between the LAB content and *Enterobacteriaceae* content were significantly negatively correlated (correlation coefficient = −0.58), which may suggest that the increase in LAB content had an influence on inhibiting of growth of *Enterobacteriaceae*. Similar observations (reduction of the number of *Enterobacteriaceae* with growth of LAB) have been described by Greppi et al [22] who monitoring of the microbiota of fermented sausages. This dependence results from the activity of LAB which can produce lactic acid (which increases the acidity of the environment) and bacteriocins (which inhibit the growth i.e. *Enterobacteriaceae*) [23].

### Influence of different type of meat on biogenic amine content in dry cured sausages

The content of BA after processing of dry-cured sausages is presented in the Table 5. Polyamines as spermine, spermidine and agmatine were not detected in any sample. Small amounts of histamine were detected in each variants of sausages. In every type of batch all detected BA content are significantly lower in the sausages made from the fallow deer meat (p<0.05). This could result from different type of meat. The first significant difference between the beef and fallow deer meat is free amino acids content. According to the literature game meat is characterized by lower content of most amino acids precursors for BA, except lysine [24–27]. Paleari et al [28] evaluated a composition of meat products from different animal species. Their results show that after the curing, fermentation and drying, the content of free amino acids (which are precursors for BA) in the deer products are significantly higher than in the beef products (p<0.05) [28]. Changes between the raw and processing products can indicate that, other factors during BA formation could be more important than the amino acids precursors [29]. Another difference that can affect BA formation, depending on the type of meat, is type and quantity of endogenous amino acid decarboxylase and its activity which can contribute to generation of BA [1]. Likewise, in different kinds of meat contaminating bacteria may belong to different species and strains, which can have various amino acid decarboxylase activity [11,30] or have ability to produce detoxifying BA—amine oxidase enzymes [11,12]. The lower level of BA in the sausages made from the fallow deer correlated well with the changes in *Enterobacteriaceae* and LAB contents. The LAB content increased more in the fallow deer sausages than in the beef sausages, also the *Enterobacteriaceae* content decreased in the higher level of whey addition in the fallow deer sausages. It confirms, the content of BA in the products is highly related with occurrence of spoilage microorganisms to which belong *Enterobacteriaceae* [11,31,32]. Pircher et al [32] also researched the formation of most important BA by bacteria isolated from the meat fermented sausages. Their results show that the LAB strains isolated from sausages are not able produce putrescine and cadaverine, but some of them can produce histamine and tyramine, which is similar to the results obtained by Pircher et al [32]. This explains the appearance of histamine in the fallow deer sausages (with the higher increase of LAB content), also the difference in the tyramine content between samples from the fallow deer and beef meat are not as large as in the case of putrescine or cadaverine content.

The most abundant BA in both cases were putrescine and cadaverine, which corresponded with the results obtained by Laranjo et al [33]. However very often as the most abundant BA in dry fermented/dry cured meat products is tyramine with cadaverine and putrescine being next most abundant, respectively [34,35].

### Influence of different additives on biogenic amine formation

The total BA content in samples with the addition of acid whey are significantly lower than in the control or reference sample. The drop in the BA content compared with the reference and the control samples were respectively 19%; 0%; 20% for AW, LAW, and 2 LAW in beef sausage samples and 51%; 31%; and 32% respectively for fallow deer sausages. The changes in the content for each BA are not as clearly visible and not all the differences are significant (p>0.05). However, the biggest impact of acid whey addition on the BA content was observed in the case of cadaverine. Each sample with the liquid acid whey addition (AW) and with the addition of double portion of freeze-dried acid whey (2LAW) had the significantly lower content of cadaverine (p<0.05), in the case of the sample LAW (freeze-dried acid whey) the significant difference was only in the fallow deer sausages (p<0.05). There were no observed significant changes in the putrescine and tyramine content dependent on the applied additive (p>0.05). The addition of organic acid whey causes a drop in a pH value and the increase content of microorganisms (mainly the LAB, which can induce further acidification), thus the growth of decarboxylating microorganisms is inhibited (by unfavourable conditions) resulting in a decreasing formation of BA’s [36]. The LAB as mentioned earlier are mainly related to the formation of tyramine and histamine [32]. Detailed research of Rzepkowska et al [16] concerned organic whey as a source of *Lactobacillus* strains with selected properties. They investigated microorganisms isolated from the organic acid whey. Results of their study shows that most of *Lactobacillus* stains present in organic acid whey can metabolise an arginine, which is the amino acid precursor for BA formation [16].

Significant lower level of total BA content in samples with the acid whey addition (p<0.05) may also result from activities of some bacterial strains occurring in acid whey. Rzepkowska et al [16] in their research isolated and identified the strains from similar organic whey as it was used at this study. Their results showed that among isolated species was *Lactobacillus plantarum*, which is one of many, with proven ability to degrade BA due to production of amine oxidase enzymes [16, 37,38]. To reduce the occurrence of BA in food the EFSA, recommends paying special attention to the hygienic quality of the raw materials and fermentation process as the most important factors for the biogenic accumulation in the fermented products [8]. Our research showed that use of acid whey can be a technological factor preventing the BA formation.

## CONCLUSION

The use of acid whey (freeze-dried and liquid) produced a decrease in *Enterobacteriaceae* content of dry fermented sausages without added nitrite. The largest decreases were observed in fallow deer sausages with the different addition of acid whey (AW, LAW, 2LAW). The BA as tyramine, putrescine and cadaverine were detected in all tested sausages. The level of total BA in beef sausages was distinctly higher than the corresponding samples from the fallow deer. The addition of acid whey (freeze-dried and liquid) caused the reduction of BA content. A larger reduction of BA was observed in samples with liquid acid whey compared to freeze dried acid whey. The results obtained here suggest that the freeze-drying can be good way to eliminate the disadvantages associated with usage of organic liquid acid whey (perishable and high water content). However further research in these areas is needed to specify the effect of acid whey microorganisms and the different amount of acid whey on BA formation.

## Figures and Tables

**Table 1:** Characteristics of acid whey

Items	pH	Lactic acid bacteria (cfu/mL)
Liquid acid whey	4.13±0.01	4.58±0.08
Freeze-drying acid whey1)	4.13±0.02	5.11±0.06

1) Acid whey powder dissolved in saline.

**Table 2:** Variants of sausages batches

Variant1)	Curing mixture	Sea salt	Glucose	Liquid ingredient
C	2.8	-	0.6	5% Water
S	-	2.8	0.6	5% Water
AW	-	2.8	0.6	5% Liquid acid whey
LAW	-	2.8	0.6	Powder after freeze-drying of 5% addition of liquid acid whey dissolved in saline to a final volume equal to 5% addition
2LAW	-	2.8	0.6	Powder after freeze-drying of 10% addition of liquid acid whey dissolved in saline to a final volume equal to 5% addition

The number of additives is given in percent relative to the mass of raw materials.

1) C, cured; S, salted; AW, sample with liquid acid whey; LAW, sample with freeze-dried acid whey; 2LAW, sample with double portion of freeze-dried acid whey.

**Table 3:** Change in lactic acid bacteria and Enterobacteriaceae content between stuff and final product (cfu/g)

Variants1)	Type of meat	ΔLAB	Δ*Enterobacteriaceae*
C	Beef	+0.19±0.08^e^	−0.90±0.11^cd^
	Fallow deer	+2.04±0.10^ab^	−1.13±0.21^bc^
S	Beef	+0.34±0.07^de^	−0.44±0.23^d^
	Fallow deer	+2.20±0.27^a^	−0.82±0.17^cd^
AW	Beef	+0.68±0.09^cd^	−0.41±0.29^d^
	Fallow deer	+1.60±0.28^b^	−1.73±0.62^ab^
LAW	Beef	+0.73±0.18^cd^	−0.74±0.18^cd^
	Fallow deer	+1.78±0.08^ab^	−2.07±0.29^a^
2LAW	Beef	+0.93±0.13^c^	−0.78±0.15^cd^
	Fallow deer	+1.96±0.11^ab^	−1.91±0.20^a^

LAB, lactic acid bacteria.

1) C, cured; S, salted; AW, sample with liquid acid whey; LAW, sample with freeze-dried acid whey; 2LAW, sample with double portion of freeze-dried acid whey.

a–f In column, sample with the same letters are not significantly different at p<0.05.

**Table 4:** Significance levels showed by experimental factors and their interactions for microbial changes and biogenic amine content of dry fermented sausages

Factor	Microbial changes		Biogenic amine content				
	
	ΔLAB	Δ*Enterobacteriaceae*	HIS	TYR	PUT	CADA	Total
Type of meat (M)	**	**	**	**	**	**	**
Variant (V)	*	**	**	**	n.s.	**	**
M×V	**	**	**	**	**	**	*

LAB, lactic acid bacteria; HIS, histidine; TYR, tyrosine; PUT, putrescine; CADA, cadaverine.

* Significance at level p<0.05;

** Significance at level p<0.01;

n.s., not significant.

**Table 5:** Biogenic amines content (mg/g product) in dry fermented sausages at the end of production process

Batch1)	Type of meat	Histamine	Tyramine	Putrescine	Cadaverine	Total
C	Beef	n.d.	0.430±0.026^ab^	0.792±0.056^a^	0.573±0.028^b^	1.794±0.109^a^
	Fallow deer	0.095±0.005^a^	0.152±0.016^c^	0.238±0.015^c^	0.347±0.013^c^	0.832±0.047^c^
S	Beef	n.d.	0.397±0.068^ab^	0.659±0.037^b^	0.750±0.082^a^	1.806±0.188^a^
	Fallow deer	0.082±0.003^b^	0.125±0.010^c^	0.255±0.017^c^	0.358±0.038^c^	0.820±0.065^c^
AW	Beef	n.d.	0.380±0.013^b^	0.753±0.033^ab^	0.315±0.009^c^	1.448±0.055^b^
	Fallow deer	0.028±0.049^d^	0.108±0.020^c^	0.183±0.020^c^	0.193±0.020^de^	0.512±0.009^d^
LAW	Beef	n.d.	0.473±0.018^a^	0.795±0.035^a^	0.527±0.003^b^	1.795±0.055^a^
	Fallow deer	0.068±0.003^c^	0.147±0.003^c^	0.203±0.008^c^	0.155±0.010^e^	0.573±0.018^d^
2LAW	Beef	n.d.	0.388±0.019^ab^	0.768±0.060^a^	0.237±0.013^cd^	1.430±0.088^b^
	Fallow deer	0.060±0.005^d^	0.155±0.005^c^	0.188±0.008^c^	0.158±0.016^e^	0.562±0.015^d^

n.d., not detected

1) C, cured; S, salted; AW, sample with liquid acid whey; LAW, sample with freeze-dried acid whey; 2LAW, sample with double portion of freeze-dried acid whey.

a–e Means with the same letters in columns are not significantly different at p<0.05.

## References

[1] Halász A, Barath A, Simon-Sarkadi L, Holzapfel W (1994). Biogenic amines and their production by microorganisms in food. Trends Food Sci Technol.

[2] Santos MS (1996). Biogenic amines: their importance in foods. Int J Food Microbiol.

[3] Lovenberg W (1973). Some vaso- and psychoactive substances in food: amines, stimulants, depressants, and hallucinogens. Toxicants occurring naturally foods.

[4] Ten Brink B, Damink C, Joosten HMLJ, In’t Veld JHJ (1990). Occurrence and formation of biologically active amines in foods. Int J Food Microbiol.

[5] Ruiz-Capillas C, Jiménez-Colmenero F (2005). Biogenic amines in meat and meat products. Crit Rev Food Sci Nutr.

[6] Byun BY, Bai X, Mah JH (2013). Occurrence of biogenic amines in *Doubanjiang* and *Tofu*. Food Sci Biotechnol.

[7] Alvarez MA, Moreno-Arribas MV (2014). The problem of biogenic amines in fermented foods and the use of potential biogenic amine-degrading microorganisms as a solution. Trends Food Sci Technol.

[8] EFSA. Panel on Biological Hazards (BIOHAZ) (2011). EFSA Scientific opinion on risk based control of biogenic amine formation in fermented foods. EFSA J.

[9] Stadnik J, Dolatowski ZJ (2010). Biogenic amines in meat and fermented meat products. Acta Sci Pol Technol Aliment.

[10] Leuschner RG, Heidel M, Hammes WP (1998). Histamine and tyramine degradation by food fermenting microorganisms. Int J Food Microbiol.

[11] Bover-Cid S, Hugas M, Izquierdo-Pulido M, Vidal-Carou MC (2001). Amino acid-decarboxylase activity of bacteria isolated from fermented pork sausages. Int J Food Microbiol.

[12] Gardini F, Özogul Y, Suzzi G, Tabanelli G, Özogul F (2016). Technological factors affecting biogenic amine content in foods: a review. Front Microbiol.

[13] Latorre-Moratalla ML, Veciana-Nogués T, Bover-Cid S (2008). Biogenic amines in traditional fermented sausages produced in selected European countries. Food Chem.

[14] Wójciak KM, Dolatowski ZJ (2015). Effect of acid whey on nitrosylmyoglobin concentration in uncured fermented sausage. LWT-Food Sci Technol.

[15] Karwowska M, Kononiuk A (2018). Addition of acid whey improves organic dry-fermented sausage without nitrite production and its nutritional value. Int J Food Sci Technol.

[16] Rzepkowska A, Zielińska D, Ołdak A, Kołożyn-Krajewska D (2017). Organic whey as a source of *Lactobacillus* strains with selected technological and antimicrobial properties. Int J Food Sci Technol.

[17] Dec B, Chojnowski W (2006). Characteristics of acid whey powder partially demineralised by nanofiltration. Pol J Food Nutr Sci.

[18] Papavasiliou G, Kourkoutas Y, Rapti A, Sipsas V, Soupioni M, Koutinas AA (2008). Production of freeze-dried kefir culture using whey. Int Dairy J.

[19] (2006). EN ISO/IEC 17025:2005 General requirements for the competence of testing and calibration laboratories.

[20] Wójciak KM, Dolatowski ZJ, Kołożyn-Krajewska D (2015). Use of acid whey and probiotic strains to improve microbiological quality and sensory acceptance of organic fermented sausage. J Food Process Preserv.

[21] Stadnik J, Stasiak DM (2016). Effect of acid whey on physicochemical characteristics of dry-cured organic pork loins without nitrite. Int J Food Sci Technol.

[22] Greppi A, Ferrocino I, La Storia A, Rantsiou K, Ercolini D, Cocolin L (2015). Monitoring of the microbiota of fermented sausages by culture independent rRNA-based approaches. Int J Food Microbiol.

[23] Woraprayote W, Malila Y, Sorapukdee S, Swetwiwathana A, Benjakul S, Visessanguan W (2016). Bacteriocins from lactic acid bacteria and their applications in meat and meat products. Meat Sci.

[24] Strazdina V, Jemeljanovs A, Sterna V, Vjazevica V (2011). Evaluation of protein composition of game meat in Latvian farms and wildlife. Agron Res.

[25] Holló G, Csapó J, Szűcs E, Tőzsér J, Repa I, Holló I (2001). Influence of breed, slaughter weight and gender on chemical composition of beef. Part 1. Amino acid profile and biological value of proteins. Asian-Austral J Anim Sci.

[26] Cygan-Szczegielniak D, Janicki B (2012). Amino acids content and basic chemical composition of roe deer (*Capreolus capreolus* L.) meat. Pol J Vet Sci.

[27] Okuskhanova E, Assenova B, Rebezov M (2017). Study of morphology, chemical, and amino acid composition of red deer meat. Vet World.

[28] Paleari MA, Moretti VM, Beretta G, Mentasti T, Bersani C (2003). Cured products from different animal species. Meat Sci.

[29] Latorre-Moratalla ML, Bover-Cid S, Bosch-Fusté J, Veciana-Nogués MT, Vidal-Carou MC (2014). Amino acid availability as an influential factor on the biogenic amine formation in dry fermented sausages. Food Control.

[30] Pons-Sánchez-Cascado S, Bover-Cid S, Veciana-Nogués MT, Vidal-Carou MC (2005). Amino acid-decarboxylase activity of bacteria isolated from ice-preserved anchovies. Eur Food Res Technol.

[31] Lorenzo JM, Cachaldora A, Fonseca S, Gómez M, Franco I, Carballo J (2010). Production of biogenic amines “*in vitro*” in relation to the growth phase by *Enterobacteriaceae* species isolated from traditional sausages. Meat Sci.

[32] Pircher A, Bauer F, Paulsen P (2007). Formation of cadaverine, histamine, putrescine and tyramine by bacteria isolated from meat, fermented sausages and cheeses. Eur Food Res Technol.

[33] Laranjo M, Gomes A, Agulheiro-Santos AC (2017). Impact of salt reduction on biogenic amines, fatty acids, microbiota, texture and sensory profile in traditional blood dry-cured sausages. Food Chem.

[34] Rabie MA, Peres C, Malcata FX (2014). Evolution of amino acids and biogenic amines throughout storage in sausages made of horse, beef and turkey meats. Meat Sci.

[35] Wójciak KM, Solska E (2016). Evolution of free amino acids, biogenic amines and N-nitrosoamines throughout ageing in organic fermented beef. Acta Sci Pol Technol Aliment.

[36] Zhang Q, Lin S, Nie X (2013). Reduction of biogenic amine accumulation in silver carp sausage by an amine-negative *Lactobacillus plantarum*. Food Control.

[37] Fadda S, Vignolo G, Oliver G (2001). Tyramine degradation and tyramine/histamine production by lactic acid bacteria and *Kocuria* strains. Biotechnol Lett.

[38] Herrero-Fresno A, Martínez N, Sánchez-Llana E (2012). *Lactobacillus casei* strains isolated from cheese reduce biogenic amine accumulation in an experimental model. Int J Food Microbiol.

